# Multiomics Mendelian Randomization Identifies Lactylation‐Related Molecular Traits in Type 1 Diabetes

**DOI:** 10.1155/jdr/9544974

**Published:** 2026-07-03

**Authors:** Yu Ding, Shiyang Gao, Lingwen Ying, Jie Tang, Guoying Chang, Yiguo Huang, Tingting Yu, Xin Li, Xiumin Wang

**Affiliations:** ^1^ Department of Endocrinology and Metabolism, Shanghai Children′s Medical Center, Shanghai Jiao Tong University School of Medicine, Shanghai, China, shsmu.edu.cn; ^2^ Department of Medical Genetics and Molecular Diagnostic Laboratory, Shanghai Children′s Medical Center, Shanghai Jiao Tong University School of Medicine, Shanghai, China, shsmu.edu.cn; ^3^ CAS Key Laboratory of Computational Biology, Shanghai Institute of Nutrition and Health, University of Chinese Academy of Sciences, Chinese Academy of Sciences, Shanghai, China, cas.cn

**Keywords:** genome-wide association study, lactylation, Mendelian randomization, multiomics, posttranslational protein modification, quantitative trait loci, type 1 diabetes

## Abstract

**Background:**

Lactylation is a posttranslational modification involved in cellular metabolic and immune processes. Although dysregulated lactate metabolism has been implicated in type 1 diabetes (T1D), the genetic relevance of lactylation‐related molecular traits remains poorly defined.

**Methods:**

Summary‐data–based Mendelian randomization (SMR) was applied to integrate blood‐derived methylation/expression/protein quantitative trait loci (mQTLs/eQTLs/pQTLs) for 353 lactylation‐related genes with a T1D genome‐wide association study (7467 cases and 10,218 controls). The HEIDI test evaluated heterogeneity. Shared genetic signals were assessed by Bayesian colocalization. Associations identified in the discovery analysis were replicated in the FinnGen_R12_T1D and FinnGen_R9_E4_DM1NEU cohorts. mQTL‐eQTL SMR analysis evaluated methylation–expression regulatory relationships. Steiger directionality testing and GTEx tissue eQTL analyses were performed for prioritized genes.

**Results:**

SMR prioritized *PDAP1*, *PNKD*, and *HMGB1* as candidate genes associated with T1D. At the DNA methylation level, methylation at cg05715492 (*PDAP1*) was positively associated with T1D risk (OR = 1.29, 95% CI: 1.09–1.53), whereas cg05991184 (*PNKD*) showed an inverse association (OR = 0.73, 95% CI: 0.59–0.90). cg22712983, cg26786924, and cg04880052 in *PNKD* exhibited positive associations. cg25251738 (*HMGB1*) methylation was inversely associated with risk (OR = 0.85, 95% CI: 0.75–0.97). At the expression level, genetically predicted *PDAP1* (OR = 0.26, 95% CI: 0.10–0.66) and *HMGB1* (OR = 0.45, 95% CI: 0.22–0.91) expressions were inversely associated with T1D risk, whereas *PNKD* expression was positively associated (OR = 1.13, 95% CI: 1.04–1.23). mQTL‐eQTL SMR analyses demonstrated significant methylation–expression associations at *PDAP1*, *PNKD*, and *HMGB1* loci. Steiger directionality testing supported the observed associations. In GTEx datasets, higher *PNKD* expression was associated with increased T1D risk in both whole blood and spleen.

**Conclusion:**

*PNKD*, *PDAP1*, and *HMGB1* were prioritized as lactylation‐related candidate genes associated with T1D risk through integrated genetic analyses. This finding generates hypothesis for future investigations into the potential involvement of lactylation‐related pathways in T1D pathogenesis.

## 1. Introduction

Type 1 diabetes (T1D) is a chronic autoimmune disease resulting from immune‐mediated destruction of insulin‐producing pancreatic *β*‐cells, leading to absolute insulin deficiency and lifelong dependence on exogenous insulin [1]. The global burden of T1D continues to increase, particularly among children and adolescents, representing a growing public health concern [2]. Although genetic susceptibility, immune dysregulation, and metabolic disturbances are well‐established contributors to T1D [3–5], the mechanisms by which genetic variation regulates metabolic and epigenetic processes involved in immune and inflammatory responses remain unclear. This gap limits the interpretation of genetic association signals and the prioritization of functional targets.

Lactylation is a posttranslational modification in which lactate‐derived lactyl groups are added to lysine residues on histone and nonhistone proteins [6]. This modification links cellular metabolic status and gene regulation or protein function, participating in immune regulation, inflammatory responses, and metabolic processes [7]. Dysregulated lactate metabolism has been implicated in T1D and its complications. For example, impaired lactate clearance and elevated circulating lactate levels have been observed in individuals with T1D [8]. Dysregulated histone lactylation has also been implicated in diabetes‐related cognitive dysfunction through neuronal gene expression and epigenetic regulation [9]. These findings suggest that lactylation may function upstream of immune and inflammatory pathways in T1D. Identifying lactylation‐related genes is therefore necessary to define the metabolic‐epigenetic mechanisms underlying T1D risk.

Genome‐wide association studies (GWAS) have identified multiple loci associated with T1D risk, but most signals map to noncoding regions [10], and their regulatory effects on downstream molecular traits remain unclear. In particular, there is a lack of genetic‐level evaluation of lactylation‐related genes in T1D. Existing studies largely focus on immune signaling or *β*‐cell function [11], whereas the potential contribution of lactylation‐related molecular regulation has received little attention. Given the confounding and reverse causation inherent in traditional observational studies [12] and the complexity of T1D pathogenesis, integrative genetic approaches are therefore required to evaluate whether genetic variation in lactylation‐related genes is associated with T1D risk. Summary‐data–based Mendelian randomization (SMR) provides a genetic framework for integrating disease‐associated variants with intermediate molecular traits at multiple omics levels [13]. Since the majority of T1D‐associated genetic variants map to noncoding regions, they likely exert their effects by regulating intermediate molecular phenotypes, such as DNA methylation, gene expression, and protein abundance (quantitative trait loci [QTLs]). Therefore, integrating GWAS data with these blood‐derived QTL datasets via SMR provides a powerful framework to systematically evaluate whether lactylation‐related molecular traits mediate T1D risk, while minimizing confounding and reverse causation [14].

To bridge the critical gap regarding the genetic relevance of lactylation in T1D etiology, this study applied an SMR‐based multiomics integration strategy to assess genetic associations between lactylation‐related molecular traits and T1D. The results prioritize candidate genes for functional validation and extend the interpretation of T1D risk loci beyond established immune pathways to lactylation‐related molecular regulation, potentially upstream of immune dysregulation.

## 2. Materials and Methods

### 2.1. Study Design

**Figure 1:**
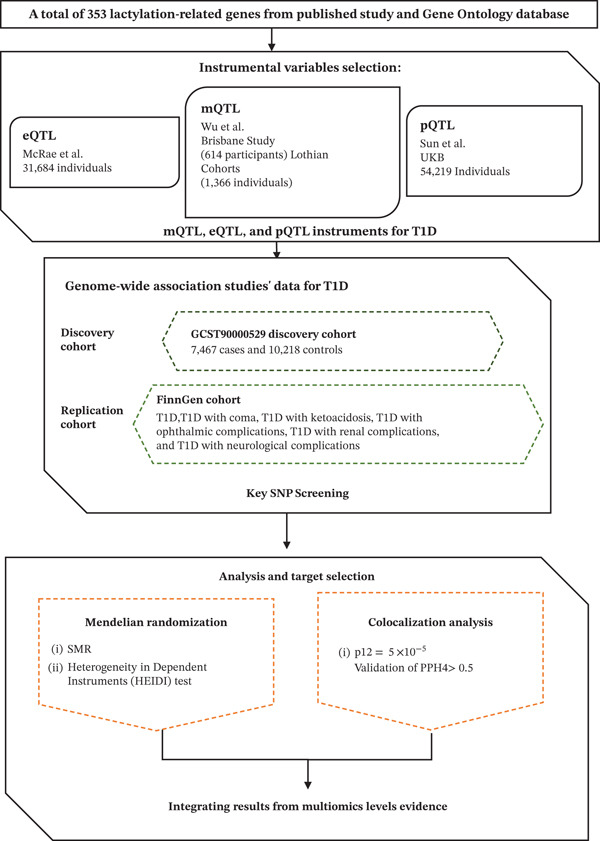
Study design: overview of the study design for identifying lactylation‐related genes associated with T1D using SMR and colocalization analyses.

This study employed a two‐sample SMR approach to evaluate associations between genetic variants influencing DNA methylation, gene expression, and protein abundance of lactylation‐related genes and the risk of T1D. A multiomics integration strategy was applied, followed by colocalization analysis to assess shared genetic signals and replication in independent cohorts. An overview of the study design is presented in Figure 1.

### 2.2. Data Sources

A comprehensive set of lactylation‐related genes was compiled from two independent sources. First, 332 genes were retrieved from a recently published study that identified a lactylation‐related gene signature [15]. Second, 25 genes associated with lactylation were identified by querying the Gene Ontology (GO) database (http://geneontology.org). After merging these datasets and removing duplicates, a final set of 353 unique lactylation‐related genes was included in the analysis. The discovery GWAS dataset for T1D was GCST90000529 [16], including 7467 T1D cases and 10,218 controls of European ancestry. Replication analyses were performed using FinnGen GWAS data, including T1D (finngen_R12_T1D; 4721 cases/403,489 controls), and T1D with neurological complications (finngen_R9_E4_DM1NEU; 1077 cases/308,280 controls; Table S1).

Blood eQTL data for lactylation‐related genes were sourced from the eQTLGen consortium (*n* = 31,684) [17]. To evaluate tissue‐specific expression associations for prioritized genes, eQTL data from the Genotype‐Tissue Expression (GTEx) project were analyzed [18]. Blood mQTL data were derived from a meta‐analysis of two European cohorts: the Brisbane Systems Genetics Study (*n* = 614) and the Lothian Birth Cohorts (*n* = 1366) [19]. Blood pQTL data were obtained from a published study including 54,219 participants of European ancestry [20]. All datasets were publicly available and deidentified. No additional ethical approval was required.

### 2.3. SMR Analysis

SMR and Heterogeneity in Dependent Instruments (HEIDI) tests were performed using the SMR software (v1.3.1) to assess the association between DNA methylation, gene expression, or protein abundance of lactylation‐related genes (exposures) and T1D risk (outcome). Cis‐QTLs were defined as SNPs located within ±1000 kb of the gene/probe and associated with the molecular trait at a *p* value of <5.0 × 10^−8^. SNPs with an allele frequency difference of > 0.2 between the QTL data, linkage disequilibrium panel, and GWAS data were excluded. The maximum proportion of SNPs allowed to have allele frequency differences was set to the default of 0.05 [21].

A multi‐SNP SMR approach was applied, incorporating all cis‐region SNPs with *p* < 5.0 × 10^−8^ and in linkage disequilibrium with the top cis‐QTL (r^2^ < 0.9). The HEIDI test was used to assess heterogeneity, and p_HEIDI > 0.01 indicates no evidence of heterogeneity due to linkage. To account for multiple testing, false discovery rate (FDR) correction was applied. Results meeting p_SMR < 0.05, p_SMR_multi < 0.05, and p_HEIDI > 0.01 were considered significant and subjected to colocalization analysis [22]. In addition to QTL‐T1D associations, mQTL (exposure)–eQTL (outcome) associations for signals overlapping between mQTL and eQTL analyses were evaluated using SMR to assess methylation–expression relationships. Prioritized genes were also evaluated using tissue‐specific GTEx eQTL datasets following the same SMR framework. The cis‐mQTL SNPs used as instrumental variables and their corresponding effects on CpG methylation and T1D risk are summarized in Table S2.

### 2.4. Colocalization Analysis

Colocalization analysis was performed using the R package coloc to assess whether the QTL and GWAS signals were driven by a shared genetic variant. The colocalization region windows were set to ±500 kb for mQTL‐GWAS and ±1000 kb for eQTL‐GWAS and pQTL‐GWAS analyses [23–25]. PPH4 represents the posterior probability that the QTL and GWAS signals are driven by a shared genetic variant. To identify highly reliable candidates, we applied a tiered colocalization screening strategy. A strict threshold of PPH4 > 0.8, which is widely adopted as standard practice for strong colocalization [26], was required for genes supported by only a single omics layer. However, for loci demonstrating convergent evidence across multiple omics layers (e.g., significant SMR associations at both methylation and expression levels), the colocalization threshold was relaxed to PPH4 > 0.5. This relaxed threshold has been considered indicative of shared genetic signals in prior studies [27] and was applied here to prioritize functionally linked multiomics targets and increase sensitivity in this exploratory analysis. Accordingly, a QTL signal and a GWAS signal were considered colocalized when PPH4 exceeded the respective tier threshold and the probability of distinct variants (PPH3) was below 0.5. Colocalization was evaluated using p12 priors of 5 × 10^−5^ or 1 × 10^−5^, which allow detection of shared signals in the presence of weaker QTL effects [28, 29]. Additional colocalization analyses were performed using p12 values from 1 × 10^−6^ to 1 × 10^−4^ to examine the influence of prior selection on the results.

### 2.5. Steiger Directionality Analysis

For prioritized QTL‐T1D associations, independent cis‐QTL variants within ±500 kb of the target locus were selected using the following criteria: *p* < 5 × 10^−8^, minor allele frequency > 0.01, F statistic > 10, and linkage disequilibrium r^2^ < 0.001 within a 10,000‐kb window. Steiger directionality testing was performed to assess the direction of the association between molecular traits and T1D risk.

### 2.6. Statistical Analyses

Statistical analyses were conducted using R (v4.3.0). Manhattan plots and forest plots were generated using the “ggplot2” and “forestplot” R packages. Locus plots and effect plots were produced as previously described [21].

## 3. Results

### 3.1. Identification of Lactylation‐Related Blood mQTL, eQTL, and pQTL Associations With T1D

**Figure 2:**
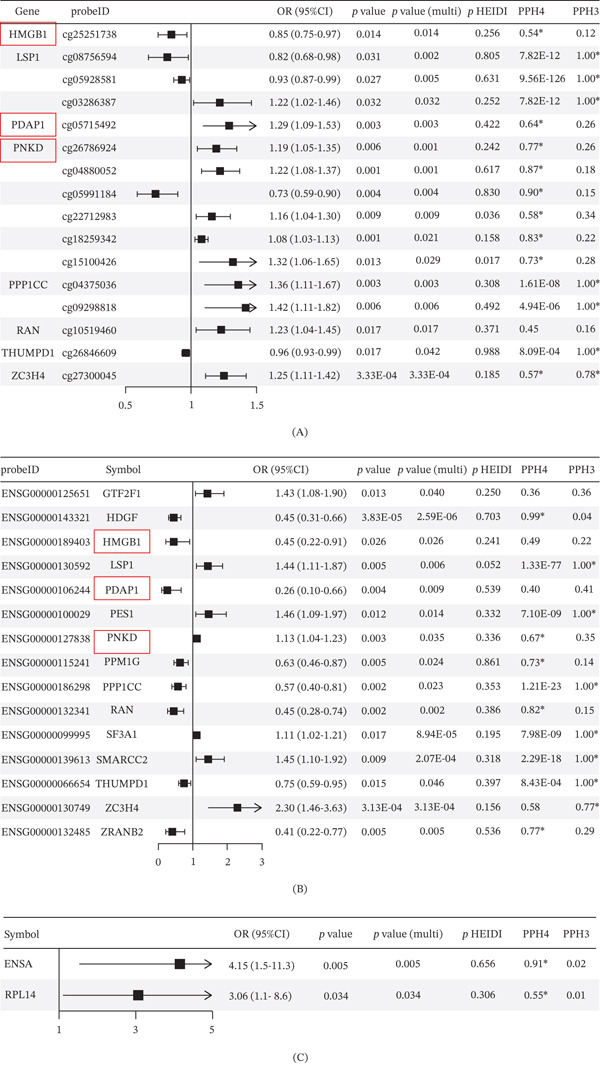
Forest plots of summary‐data–based Mendelian randomization (SMR) associations between lactylation‐related molecular traits and type 1 diabetes (T1D) risk in the GCST90000529 discovery dataset. Panels show results based on (A) DNA methylation quantitative trait loci (mQTLs), (B) gene expression quantitative trait loci (eQTLs), and (C) protein quantitative trait loci (pQTLs). Only associations meeting p_SMR < 0.05, p_SMR_multi < 0.05, and p_HEIDI > 0.01 are presented. Genes prioritized for downstream analyses (*PNKD*, *PDAP1*, and *HMGB1*) are highlighted with red boxes. Odds ratios (ORs) and 95% confidence intervals (CIs) are displayed. Squares represent ORs; horizontal lines indicate 95% CIs. PPH4 and PPH3 denote posterior probabilities for a shared causal variant and distinct causal variants, respectively, from Bayesian colocalization analysis. An asterisk (∗) indicates PPH4 > 0.5.

In the discovery phase, SMR analysis identified 51 CpG sites mapping to 33 lactylation‐related genes associated with T1D risk. Among these, 20 CpG sites corresponding to 10 genes showed colocalization evidence (Table S3). At the gene expression level, SMR analysis of blood eQTL data identified 15 lactylation‐related genes associated with T1D risk, of which five (*PPM1G*, *HDGF*, *ZRANB2*, *RAN*, and *PNKD*) were supported by colocalization evidence (Table S4). Eight genes overlapped between the mQTL‐ and eQTL‐based results, including *HMGB1*, *LSP1*, *PDAP1*, *PNKD*, *PPP1CC*, *RAN*, *THUMPD1*, and *ZC3H4*. A forest plot summarizing mQTL–T1D associations for these overlapping genes is shown in Figure 2A. The eQTL‐based SMR results for all 15 identified genes are presented in Figure 2B.

At the protein level, SMR analysis identified two lactylation‐related proteins with genetically predicted blood abundance associated with T1D risk. Higher levels of ENSA (OR = 4.15, 95% CI: 1.5–11.3) and RPL14 (OR = 3.06, 95% CI: 1.1–8.6) were associated with increased T1D risk, both with colocalization support (Table S5; Figure 2C). These pQTL signals did not overlap with mQTL or eQTL results, likely due to limited pQTL coverage (41 genes). Based on our tiered screening strategy, Table 1 summarizes the prioritized genes falling into two distinct categories: (1) single‐omics loci with strong colocalization evidence (PPH4 > 0.8), such as *ENSA* (pQTL PPH4 = 0.91) and *HDGF* (eQTL PPH4 = 0.99); and (2) multiomics loci demonstrating convergent SMR associations across at least two QTL layers. For the latter, integrative analysis prioritized *HMGB1*, *PDAP1*, *PNKD*, and *RAN* as genes supported at both the methylation and expression levels, with moderate to strong colocalization evidence (PPH4 > 0.5) at least one QTL level, indicating robust cross‐layer biological relevance despite slightly relaxed individual colocalization thresholds (Table 1). The Manhattan plots display the genome‐wide distribution of blood QTL‐based SMR associations with T1D (Figure S1).

**Table 1:** Multilevel blood QTL associations of lactylation‐related genes with T1D risk.

Gene	QTL level	CpG site/probe ID	OR (95% CI)	p_SMR	p_SMR_multi	p_HEIDI	PPH4	PPH3
**HMGB1**	mQTL	cg25251738	0.85 (0.75–0.97)	0.014	0.014	0.256	*0.54* ^∗^	*0.12* ^∗^
	eQTL	ENSG00000189403	0.45 (0.22–0.91)	0.026	0.026	0.241	0.49	0.22
**PDAP1**	mQTL	cg05715492	1.29 (1.09–1.53)	0.003	0.003	0.422	*0.64* ^∗^	*0.26* ^∗^
	eQTL	ENSG00000106244	0.26 (0.10–0.66)	0.004	0.009	0.539	0.4	0.41
**PNKD**	mQTL	cg05991184	0.73 (0.59–0.9)	0.003855347	0.003855347	0.83033	*0.90* ^∗^	*0.15* ^∗^
		cg04880052	1.22 (1.08–1.37)	0.001402622	0.001402622	0.61658	*0.87* ^∗^	*0.18* ^∗^
		cg26786924	1.19 (1.05–1.35)	0.006	0.001	0.242	*0.77* ^∗^	*0.26* ^∗^
		cg18259342	1.08 (1.03–1.13)	0.00148014	0.02125634	0.15751	*0.83* ^∗^	*0.22* ^∗^
		cg22712983	1.16 (1.04–1.3)	0.008596752	0.008596752	0.03648	*0.58* ^∗^	*0.34* ^∗^
		cg15100426	1.32 (1.06–1.65)	0.01278419	0.02913028	0.01695	*0.73* ^∗^	*0.28* ^∗^
	eQTL	ENSG00000127838	1.13 (1.04–1.23)	0.003	0.035	0.336	*0.67* ^∗^	*0.35* ^∗^
LSP1	mQTL	cg08756594	0.82 (0.68–0.98)	0.031	0.002	0.805	7.82E‐12	1.00
	eQTL	ENSG00000130592	1.44 (1.11–1.87)	0.005	0.006	0.052	1.33E‐77	1.00
PPP1CC	mQTL	cg04375036	1.36 (1.11–1.67)	0.003	0.003	0.308	1.61E‐08	1.00
	eQTL	ENSG00000186298	0.57 (0.40–0.81)	0.002	0.023	0.353	1.21E‐23	1.00
RAN	mQTL	cg10519460	1.23 (1.04–1.45)	0.017	0.017	0.371	0.45	0.16
	eQTL	ENSG00000132341	0.45 (0.28–0.74)	0.002	0.002	0.386	*0.82* ^∗^	*0.15* ^∗^
THUMPD1	mQTL	cg26846609	0.96 (0.93–0.99)	0.017	0.042	0.988	8.09E‐04	1.00
	eQTL	ENSG00000066654	0.75 (0.59–0.95)	0.015	0.046	0.397	8.43E‐04	1.00
ZC3H4	mQTL	cg27300045	1.25 (1.11–1.42)	3.33E‐04	3.33E‐04	0.185	0.57 ^∗^	0.78
	eQTL	ENSG00000130749	2.30 (1.46–3.63)	3.13E‐04	3.13E‐04	0.156	0.58 ^∗^	0.77
GTF2F1	eQTL	ENSG00000125651	1.43 (1.08–1.90)	0.013	0.04	0.25	0.36	0.36
HDGF	eQTL	ENSG00000143321	0.45 (0.31–0.66)	3.83E‐05	2.59E‐06	0.703	*0.99* ^∗^	*0.04* ^∗^
PES1	eQTL	ENSG00000100029	1.46 (1.09–1.97)	0.012	0.014	0.332	7.10E‐09	1
PPM1G	eQTL	ENSG00000115241	0.63 (0.46–0.87)	0.005	0.024	0.861	*0.73* ^∗^	*0.14* ^∗^
SF3A1	eQTL	ENSG00000099995	1.11 (1.02–1.21)	0.017	8.94E‐05	0.195	7.98E‐09	1.00
SMARCC2	eQTL	ENSG00000139613	1.45 (1.10–1.92)	0.009	2.07E‐04	0.318	2.29E‐18	1.00
ZRANB2	eQTL	ENSG00000132485	0.41 (0.22–0.77)	0.005	0.005	0.536	*0.77* ^∗^	*0.29* ^∗^
ENSA	pQTL	—	4.15 (1.5–11.3)	0.005	0.005	0.656	*0.91* ^∗^	*0.02* ^∗^
RPL14	pQTL	—	3.06 (1.1–8.6)	0.034	0.034	0.306	*0.55* ^∗^	*0.01* ^∗^

*Note:* An asterisk (∗) and Italic text indicate colocalization evidence (PPH4 > 0.5 and PPH3 < 0.5). Bold text indicates genes supported at both the methylation and expression levels with colocalization evidence at least one QTL level.

### 3.2. Replication in FinnGen Cohorts

At the mQTL level (Table S6), six CpG sites were replicated in the FinnGen_R12_T1D cohort, including cg14876906 (*SSB*), cg10277195 (*MDC1*), cg22797644 (*HMGA1*), cg16499656 (*IKZF1*), and two sites in *TSSC4* (cg24752563 and cg10172068). In the FinnGen_R9_E4_DM1NEU cohort, cg12633102 (*SFPQ*), cg10277195 (*MDC1*), and cg22797644 (*HMGA1*) were replicated. Effect directions were consistent with those observed in the discovery cohort for all replicated sites, except for cg22797644 (*HMGA1*). However, these loci did not show colocalization support in the discovery analysis and were therefore not prioritized for downstream interpretation.

At the eQTL level (Table S7), *LSP1* was replicated in the FinnGen_R12_T1D cohort, and *PPM1G* was replicated in the FinnGen_R9_E4_DM1NEU cohort, with effect directions concordant with the discovery results. However, *PPM1G* was identified only at the eQTL level, whereas *LSP1* lacked colocalization support in the discovery analysis. Therefore, neither gene was prioritized for downstream interpretation. At the pQTL level, no associations were replicated in either cohort (Table S8).

### 3.3. Integration of Blood mQTL and eQTL Signals Associated With T1D

Eight genes overlapped between the significant mQTL‐T1D and eQTL‐T1D SMR results, suggesting that DNA methylation may regulate gene expression and thereby influence T1D risk. To test this hypothesis, SMR analysis was performed using blood mQTLs as exposures and eQTLs as outcomes. The full results are presented in Table S9. Eight loci demonstrated significant associations with gene expression, including cg25251738 in *HMGB1*, cg05715492 in *PDAP1*, four CpG sites in *PNKD* (cg05991184, cg22712983, cg26786924, and cg04880052), cg08756594 in *LSP1*, and cg09298818 in *PPP1CC* (Table 2). Considering the associations at both the methylation and expression levels and colocalization support at least one QTL level, *PDAP1*, *PNKD*, and *HMGB1* were prioritized for downstream interpretation. Steiger directionality analyses supported the inferred exposure‐to‐T1D direction for the prioritized signals (Table S10). Sensitivity analyses using alternative p12 priors showed that the prioritized signals retained colocalization support under the p12 values used in the primary analysis (Figure S2 and Tables S11–S12).

**Table 2:** Significant mQTL–eQTL associations for lactylation‐related genes identified by SMR analysis.

Expo_ID	Outco_Gene	p_SMR	p_SMR_multi	p_HEIDI	OR_SMR (95% CI)
cg25251738	HMGB1	5.05E‐10	5.05E‐10	0.02	1.19 (1.13–1.26)
cg05715492	PDAP1	4.46E‐06	4.46E‐06	0.171	0.86 (0.81–0.92)
cg05991184	PNKD	5.78E‐12	5.78E‐12	0.526	0.11 (0.06–0.21)
cg22712983	PNKD	1.54E‐33	1.54E‐33	0.136	3.75 (3.03–4.65)
cg26786924	PNKD	5.51E‐28	2.04E‐27	0.519	4.2 (3.25–5.43)
cg04880052	PNKD	1.34E‐30	1.34E‐30	0.024	3.66 (2.93–4.57)
cg08756594	LSP1	2.28E‐14	9.90E‐15	0.089	0.64 (0.57–0.72)
cg09298818	PPP1CC	1.39E‐08	1.39E‐08	0.184	0.56 (0.46–0.69)

Among the prioritized genes, genetically predicted *PNKD* expression was positively associated with T1D risk in both whole blood (OR = 1.26, 95% CI: 1.07–1.48) and spleen (OR = 1.20, 95% CI: 1.05–1.37) GTEx eQTL datasets, consistent with the direction observed in the discovery analysis. No eligible GTEx tissue eQTL signals were available for *PDAP1* or *HMGB1* (Table S13).

### 3.4. Hypothesized Methylation‐Expression Regulatory Mechanisms at T1D‐Associated Loci

**Figure 3:**
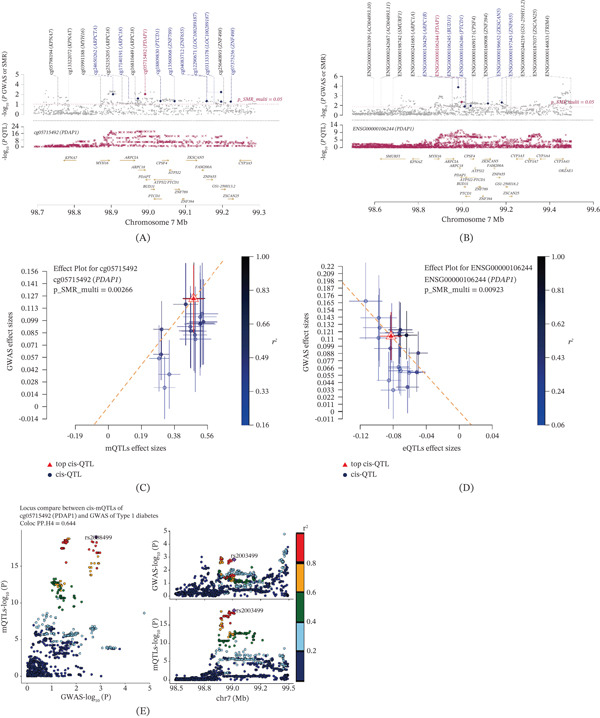
SMR and colocalization analyses for *PDAP1*. (A) Regional association plot showing the cis‐mQTL signal for cg05715492 at the *PDAP1* locus and its corresponding association with type 1 diabetes (T1D). (B) Regional association plot showing the cis‐eQTL signal for *PDAP1* expression and its corresponding association with T1D. In regional plots, the lead variant is indicated by a purple diamond, and surrounding variants are colored according to linkage disequilibrium (LD; r^2^) with the lead variant. Gene annotations are shown in the lower panel. (C) Effect size plot for the association between cg05715492 methylation (mQTL) and T1D risk. (D) Effect size plot for the association between *PDAP1* expression (eQTL) and T1D risk. Each point represents a genetic variant. Colors indicate LD (r^2^) with the top cis‐QTL variant. The red triangle denotes the top cis‐QTL variant. (E) Bayesian colocalization analysis between the cis‐mQTL of cg05715492 and the T1D genome‐wide association study (GWAS) signal. SMR, summary‐data–based Mendelian randomization; mQTL, methylation quantitative trait locus; eQTL, expression quantitative trait locus; GWAS, genome‐wide association study; LD, linkage disequilibrium. Significant SMR associations satisfied p_SMR < 0.05, p_SMR_multi < 0.05, and p_HEIDI > 0.01.

For *PDAP1*, the mQTL‐GWAS and eQTL‐GWAS association signals are shown in the locus plots (Figure 3A,B). Genetically predicted methylation at cg05715492 was positively associated with T1D risk (OR = 1.29, 95% CI: 1.09–1.53; Figure 3C), whereas genetically predicted *PDAP1* expression was inversely associated with T1D risk (OR = 0.26, 95% CI: 0.10–0.66; Figure 3D). In addition, cg05715492 methylation, which showed colocalization evidence with the T1D GWAS signal (Figure 3E), was inversely associated with *PDAP1* expression (OR = 0.86, 95% CI: 0.81–0.92; Table 2). These results suggest that higher cg05715492 methylation is associated with lower *PDAP1* expression and increased T1D risk.

**Figure 4:**
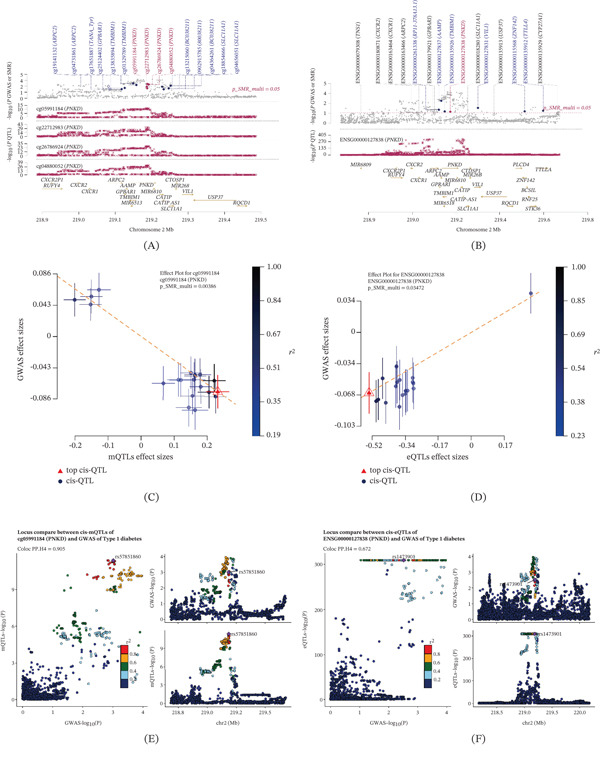
SMR and colocalization analyses for *PNKD*. (A) Regional association plot showing the cis‐mQTL signals for cg05991184, cg22712993, cg26786924, and cg04888052 at the *PNKD* locus and their corresponding associations with type 1 diabetes (T1D). (B) Regional association plot showing the eQTL signal for *PNKD* expression and its corresponding association with T1D. In regional plots, the lead variant is indicated by a purple diamond, and surrounding variants are colored according to linkage disequilibrium (LD; r^2^) with the lead variant. Gene annotations are shown in the lower panel. (C) Effect size plot for the association between cg05991184 methylation (mQTL) and T1D risk. (D) Effect size plot for the association between *PNKD* expression (eQTL) and T1D risk. Each point represents a genetic variant, with colors indicating LD (r^2^) with the top cis‐QTL variant. The red triangle denotes the top cis‐QTL variant. (E) Bayesian colocalization analysis between the cis‐mQTL of cg05991184 and the T1D genome‐wide association study (GWAS) signal. (F) Bayesian colocalization analysis between the cis‐eQTL of PNKD and the T1D GWAS signal. In colocalization plots, point colors indicate LD (r^2^) with the lead variant. SMR, summary‐data–based Mendelian randomization; mQTL, methylation quantitative trait locus; eQTL, expression quantitative trait locus; GWAS, genome‐wide association study; LD, linkage disequilibrium. Significant SMR associations satisfied p_SMR < 0.05, p_SMR_multi < 0.05, and p_HEIDI > 0.01.

The locus plots for *PNKD* mQTL‐GWAS and eQTL‐GWAS associations are shown in Figure 4A,B. To further explore the heterogeneous regulatory effects at this locus, we annotated the genomic positions of the T1D‐associated CpG sites (Table S14). Methylation at cg05991184, located 1–5 kb upstream of the transcription start site, was inversely associated with T1D risk (OR = 0.73, 95% CI: 0.59–0.90; Figure 4C), whereas methylation at promoter regions (OR = 1.16, 95% CI: 1.04–1.30), cg26786924 (OR = 1.19, 95% CI: 1.05–1.35), and an intronic region (cg04880052, OR = 1.22, 95% CI: 1.08–1.37) was positively associated with risk. At the expression level, genetically predicted *PNKD* expression was positively associated with T1D risk (OR = 1.13, 95% CI: 1.04–1.23; Figure 4D). Both mQTL and eQTL associations were supported by colocalization analyses (Figure 4E,F). mQTL‐eQTL SMR analysis demonstrated locus‐specific regulatory effects corresponding to these genomic regions. Methylation at the upstream site (cg05991184) was inversely associated with *PNKD* expression (OR = 0.11, 95% CI: 0.06–0.21), whereas methylation at the promoter (cg22712983, OR = 3.75, 95% CI: 3.03–4.65), cg26786924 (OR = 4.20, 95% CI: 3.25–5.43), and intronic sites (cg04880052, OR = 3.66, 95% CI: 2.93–4.57) was positively associated with expression (Table 2). Together, these findings suggest that methylation at cg05991184 is associated with lower *PNKD* expression and reduced T1D risk, whereas methylation at cg22712983, cg26786924, and cg04880052 is associated with higher *PNKD* expression and increased T1D risk.

**Figure 5:**
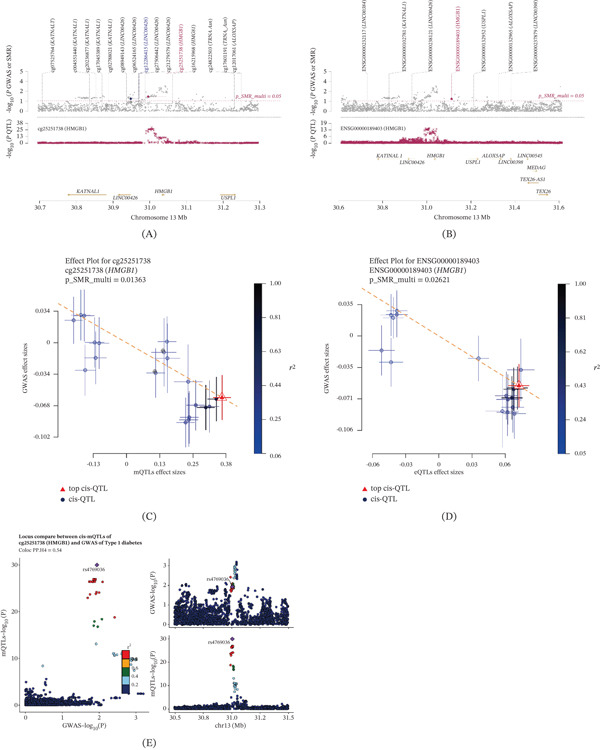
SMR and colocalization analyses for *HMGB1*. (A) Regional association plot showing the cis‐mQTL signals for cg25251738 at the *HMGB1* locus and its corresponding association with type 1 diabetes (T1D). (B) Regional association plot showing the eQTL signal for *HMGB1* expression and its corresponding association with T1D. In regional plots, the lead variant is indicated by a purple diamond, and surrounding variants are colored according to linkage disequilibrium (LD; r^2^) with the lead variant. Gene annotations are shown in the lower panel. (C) Effect size plot for the association between cg25251738 methylation (mQTL) and T1D risk. (D) Effect size plot for the association between *HMGB1* expression (eQTL) and T1D risk. Each point represents a genetic variant, with colors indicating LD (r^2^) with the top cis‐QTL variant. The red triangle denotes the top cis‐QTL variant. (E) Bayesian colocalization analysis between the cis‐mQTL of cg25251738 and the T1D genome‐wide association study (GWAS) signal. In colocalization plots, point colors indicate LD (r^2^) with the lead variant. SMR, summary‐data–based Mendelian randomization; mQTL, methylation quantitative trait locus; eQTL, expression quantitative trait locus; GWAS, genome‐wide association study; LD, linkage disequilibrium. Significant SMR associations satisfied p_SMR < 0.05, p_SMR_multi < 0.05, and p_HEIDI > 0.01.

The locus plots illustrating *HMGB1* mQTL‐GWAS and eQTL‐GWAS association signals are shown in Figure 5A,B. Methylation at cg25251738 was inversely associated with T1D risk (OR = 0.85, 95% CI: 0.75–0.97; Figure 5C). At the expression level, genetically predicted *HMGB1* expression was also inversely associated with T1D risk (OR = 0.45, 95% CI: 0.22–0.91; Figure 5D). The mQTL‐GWAS association was supported by colocalization evidence (Figure 5E). mQTL‐eQTL SMR analysis further showed that methylation at cg25251738 was positively associated with *HMGB1* expression (OR = 1.19, 95% CI: 1.13–1.26; Table 2). These findings suggest that cg25251738 methylation is associated with higher *HMGB1* expression and reduced T1D risk.

## 4. Discussion

This study identified *PNKD*, *PDAP1*, and *HMGB1* as lactylation‐related candidate genes associated with T1D through integrated genetic analyses of DNA methylation and gene expression traits. These findings provide genetic evidence supporting the involvement of lactylation‐related molecular traits in T1D and prioritize candidate loci for future functional investigation.

*PNKD* encodes the paroxysmal nonkinesigenic dyskinesia protein and has been primarily studied in neurological disorders [30]. More recently, *PNKD* has been identified in lactate metabolism‐related gene signatures and shown to promote lactate accumulation by activating PI3K‐AKT‐mTOR signaling in clear cell renal cell carcinoma [31, 32]. Given that intracellular lactate serves as a substrate for protein lactylation, these findings suggest a potential link between *PNKD* and lactylation‐related biological processes. Although *PNKD* has not previously been implicated in T1D, our results reveal that its epigenetic regulation is highly complex and dependent on the genomic location of the CpG sites (Table S14). As noted, methylation at upstream sites represses *PNKD* expression, whereas methylation at promoter or intronic regions enhances it. Biologically, methylation at promoter‐associated CpG sites may influence transcription initiation, whereas methylation at upstream or intronic sites may affect more distal regulatory elements. However, genomic location alone is unlikely to fully explain the observed bidirectional associations, and the underlying mechanisms require functional investigation.

*PDAP1* encodes platelet‐derived growth factor (PDGF)–associated protein 1, an RNA‐binding phosphoprotein that regulates stress‐resistant translation, immune cell proliferation, and growth‐factor signaling [33–35]. SMR analyses showed that higher methylation at cg05715492 was associated with reduced *PDAP1* expression and increased T1D risk, suggesting a potential relationship between DNA methylation, PDAP1 expression, and T1D susceptibility. Although *PDAP1* has not previously been implicated in T1D, recent evidence has indicated that *PDAP1* undergoes lactylation and promotes NLRP3 inflammasome activation and IL‐1*β* release [36]. Given the established role of NLRP3 inflammasome signaling and IL‐1*β* in T1D pathogenesis [37], *PDAP1* may represent a potential link between lactylation‐associated signaling and T1D susceptibility.

*HMGB1* encodes high mobility group box 1, a chromatin‐binding protein that functions as an extracellular damage‐associated molecular pattern [38]. In this study, lower methylation at cg25251738 was associated with reduced *HMGB1* expression in blood and increased T1D risk, which appears paradoxical given its proinflammatory role and suggests a complex relationship between *HMGB1* regulation and disease susceptibility. Notably, *HMGB1* has been reported to undergo lysine lactylation, which promotes its cytoplasmic translocation and extracellular release in activated macrophages [39, 40]. Extracellular *HMGB1* activates Toll‐like receptor signaling and contributes to autoimmune inflammation, including T1D [41], and HMGB1 lactylation has also been implicated in diabetic kidney disease [42].

DNA methylation may represent an important molecular layer linking genetic variation to T1D‐associated molecular traits. For *PNKD*, *PDAP1*, and *HMGB1*, mQTL‐eQTL integration identified associations between DNA methylation and gene expression. Further studies are required to determine whether the observed associations are relevant to lactylation‐related processes and T1D pathogenesis. Although the present findings are not directly translatable to clinical practice, the prioritized loci may help guide future biomarker discovery and mechanistic studies. If validated in disease‐relevant tissues and clinically accessible samples, these molecular traits could contribute to earlier identification of individuals at increased T1D risk or support the development of targeted therapeutic strategies.

A principal strength of this study is the application of mQTL‐eQTL integration to investigate associations between DNA methylation, gene expression, and T1D susceptibility at the *PNKD*, *PDAP1*, and *HMGB1* loci. Several limitations warrant careful consideration. First, although SMR and HEIDI analyses help reduce confounding due to linkage and reverse causation, they cannot completely exclude horizontal pleiotropy. Some genetic variants may influence T1D through biological pathways independent of the methylation or expression traits examined in this study. Therefore, the observed methylation–expression–T1D relationships should be interpreted as genetically supported associations rather than definitive evidence of causal mediation. Although complementary statistical approaches like MR‐Egger or CAUSE may provide additional genetic evidence, future functional investigations including in vivo animal models, in vitro mechanistic assays, and targeted clinical validation cohorts are absolutely essential to confirm the proposed regulatory directions and establish true biological causality.

Second, protein‐level validation was limited. Although pQTL analysis identified ENSA and RPL14 as T1D‐associated proteins, the available pQTL dataset covered only a small proportion of the lactylation‐related genes evaluated in this study. Consequently, the prioritized genes could not be assessed at the protein level. Future studies should validate the methylation and expression patterns of *PNKD*, *PDAP1*, and *HMGB1* in independent T1D cohorts and examine whether corresponding changes can be detected at the protein level using targeted proteomic approaches. Subsequent studies could assess the utility of these proteins as serum biomarkers for identifying individuals at high risk of T1D.

Third, our analysis relied on gene abundance as a proxy for lactylation‐related biology. We could not directly quantify protein lactylation or identify specific modification sites due to the lack of site‐specific proteomic data. In addition, colocalization was evaluated using a relatively liberal threshold (PPH4 > 0.5). Although sensitivity analyses using alternative p12 priors yielded consistent results for the prioritized loci, the colocalization findings should be interpreted as supportive.

Fourth, our analyses relied primarily on blood‐derived QTL datasets. Because T1D is characterized by immune‐mediated destruction of pancreatic *β*‐cells within the pancreatic islets [1], regulatory effects identified in blood may not fully reflect those operating in disease‐relevant tissues. To partially address this issue, we performed GTEx tissue eQTL analyses. However, eligible tissue‐specific signals were only available for *PNKD*. Notably, GTEx only provides bulk pancreas data, where islet‐specific regulatory signals are heavily diluted by dominant exocrine tissues, necessitating specialized isolated islet eQTL resources. We did not evaluate pancreatic islet eQTL resources such as TIGER [43] or InsPIRE [44], which primarily focus on T2D‐associated islet biology. Future studies integrating T1D‐relevant pancreatic islet transcriptomic, epigenomic, and eQTL datasets will be important for determining whether the associations observed in blood are preserved in disease‐relevant tissues.

Finally, all QTL and GWAS datasets included in this study were derived primarily from individuals of European ancestry. Given that T1D incidence varies significantly across global regions, the distinct genetic architectures and unique gene‐environment interactions characteristic of non‐European populations remain unrepresented in current multiomics data. Differences in allele frequencies, linkage disequilibrium structure, and genetic effect sizes across populations may alter the strength and significance of QTL‐trait associations. As a result, some multiomics signals identified in this study may exhibit different effect estimates or *p* values in other ancestral groups, potentially affecting the prioritization of candidate loci. Validation in diverse populations will be important to determine the robustness and generalizability of the prioritized loci.

## 5. Conclusion

In conclusion, this study identified *PNKD*, *PDAP1*, and *HMGB1* as lactylation‐related candidate genes associated with T1D through integrated analyses of DNA methylation, gene expression, and genetic association data. These findings provide genetic evidence linking lactylation‐related molecular traits to T1D susceptibility and highlight potential roles for epigenetic regulation in disease risk. Further functional investigations including in vivo animal models, in vitro mechanistic assays, and targeted clinical validation cohorts are required to confirm the proposed regulatory directions, validate these associations, and establish their true relevance to T1D pathogenesis.

## Author Contributions

Yu Ding and Shiyang Gao conducted the studies, collected data, and drafted the manuscript. Xiumin Wang, Xin Li, and Tingting Yu performed the statistical analysis and participated in its design. Lingwen Ying, Guoying Chang, Yiguo Huang, and Jie Tang participated in the acquisition, analysis, or interpretation of data and drafted the manuscript. Yu Ding and Shiyang Gao contributed equally to this work.

## Funding

This study was supported by Youth Clinical Cadre Hospital Fund of Shanghai Children′s Medical Center (QN‐SCMC2023‐5); National Nature Science Foundation of China (82170910); 2024‐National Clinical Key Specialty Construction Project (10000015Z155080000004); Special Fund for People′s Livelihood Scientific Research of Public Institutions under the Science and Technology Development Fund of Pudong New Area (PKJ2025‐Y03); and Special Research Project of National Health Commission Capacity Building and Continuing Education Center (GWJJZX20251003002).

## Disclosure

All authors read and approved the final manuscript.

## Ethics Statement

The authors have nothing to report.

## Consent

The authors have nothing to report.

## Conflicts of Interest

The authors declare no conflicts of interest.

## Supporting Information

Additional supporting information can be found online in the Supporting Information section.

## Supporting information


**Supporting Information 1** Figure S1: Manhattan plots of SMR associations between lactylation‐related molecular traits and T1D risk in the discovery dataset (GCST90000529). Panels display genome‐wide SMR results based on (A) mQTLs, (B) eQTLs, and (C) pQTLs. Each point represents an SMR association, plotted as −log10(p). The dashed horizontal line indicates the SMR multiple‐testing threshold (p_SMR_multi = 0.05). Selected loci of interest are annotated.

**Supporting Information 2** Figure S2: Sensitivity analysis of colocalization results under different p12 priors. (A) Sensitivity analysis of mQTL‐GWAS colocalization results for prioritized CpG sites in *PNKD*, *PDAP1*, and *HMGB1* using a ±500‐kb genomic window. (B) Sensitivity analysis of eQTL‐GWAS colocalization results for prioritized transcripts in *PNKD*, *PDAP1*, and *HMGB1* using a ±1000‐kb genomic window. Colocalization analyses were repeated using five p12 priors (1 × 10^−6^, 5 × 10^−6^, 1 × 10^−5^, 5 × 10^−5^, and 1 × 10^−4^). Heatmaps display posterior probabilities for the four coloc hypotheses: PPH1, association with the QTL trait only; PPH2, association with T1D only; PPH3, association with both traits driven by distinct causal variants; and PPH4, association with both traits driven by a shared causal variant. Numerical values represent posterior probabilities. Warmer colors indicate higher posterior probabilities. Cells outlined in gold and marked with an asterisk denote colocalization support (PPH4 > 0.5 and PPH3 < 0.5). Abbreviations: mQTL, methylation quantitative trait locus; eQTL, expression quantitative trait locus; GWAS, genome‐wide association study; T1D, type 1 diabetes; PPH, posterior probability of hypothesis.

**Supporting Information 3** Table S1: Characteristics of type 1 diabetes GWAS datasets.

**Supporting Information 4** Table S2: Characteristics of the top cis‐mQTL SNPs used as instrumental variables for key CpG sites.

**Supporting Information 5** Table S3: SMR results for associations between blood mQTLs of lactylation‐related genes and T1D in the GCST90000529 dataset. Results meeting SMR significance criteria are shown in bold (p_SMR < 0.05, p_SMR_multi < 0.05, and p_HEIDI > 0.01). Results with colocalization evidence are highlighted in red (PPH4 > 0.5 and PPH3 < 0.5).

**Supporting Information 6** Table S4: SMR results for associations between blood eQTLs of lactylation‐related genes and T1D in the GCST90000529 dataset. Results meeting SMR significance criteria are shown in bold (p_SMR < 0.05, p_SMR_multi < 0.05, and p_HEIDI > 0.01). Results with colocalization evidence are highlighted in red (PPH4 > 0.5 and PPH3 < 0.5).

**Supporting Information 7** Table S5. SMR results for associations between blood pQTLs of lactylation‐related genes and T1D in the GCST90000529 dataset. pQTLs meeting SMR (p_SMR < 0.05, p_SMR_multi < 0.05, and p_HEIDI > 0.01) and colocalization criteria (PPH4 > 0.5 and PPH3 < 0.5) are highlighted in red.

**Supporting Information 8** Table S6: SMR results for associations between blood mQTLs of lactylation‐related genes and T1D in the FinnGen_R12_T1D and Finngen R8_E4_DM1NEU cohorts. Results with p_SMR < 0.05 are shown. Signals replicated from the discovery cohort are highlighted in bold.

**Supporting Information 9** Table S7: SMR results for associations between blood eQTLs of lactylation‐related genes and T1D in the FinnGen_R12_T1D and Finngen R8_E4_DM1NEU cohorts. Results with p_SMR < 0.05 are shown. Signals replicated from the discovery cohort are highlighted in bold.

**Supporting Information 10** Table S8: SMR results for associations between blood pQTLs of lactylation‐related genes and T1D in the FinnGen_R12_T1D and Finngen R8_E4_DM1NEU cohorts. Results with p_SMR < 0.05 are shown.

**Supporting Information 11** Table S9: SMR results for mQTL‐eQTL associations of lactylation‐related genes. CpG sites significant in both mQTL‐T1D and mQTL‐eQTL analyses are highlighted in red (p_SMR < 0.05, p_SMR_multi < 0.05, and p_HEIDI > 0.01).

**Supporting Information 12** Table S10: Steiger directionality analysis of prioritized mQTL and eQTL associations with T1D risk.

**Supporting Information 13** Table S11: Colocalization sensitivity analysis of prioritized mQTL signals under different p12 priors.

**Supporting Information 14** Table S12: Colocalization sensitivity analysis of prioritized eQTL signals under different p12 priors.

**Supporting Information 15** Table S13: SMR analysis of prioritized genes using GTEx tissue eQTL datasets.

**Supporting Information 16** Table S14: Genomic annotations and regional positions of type 1 diabetes (T1D)–associated CpG sites at the *PNKD* locus.

**Supporting Information 17**  

## Data Availability

All data generated or analyzed during this study are included in this published article.
