# Supplementary figures and images for: Multiomics Mendelian Randomization Identifies Lactylation‐Related Molecular Traits in Type 1 Diabetes
Source: J Diabetes Res. 2026 Jul 3;2026:9544974. doi: 10.1155/jdr/9544974 (PMC13329454; doi:10.1155/jdr/9544974)

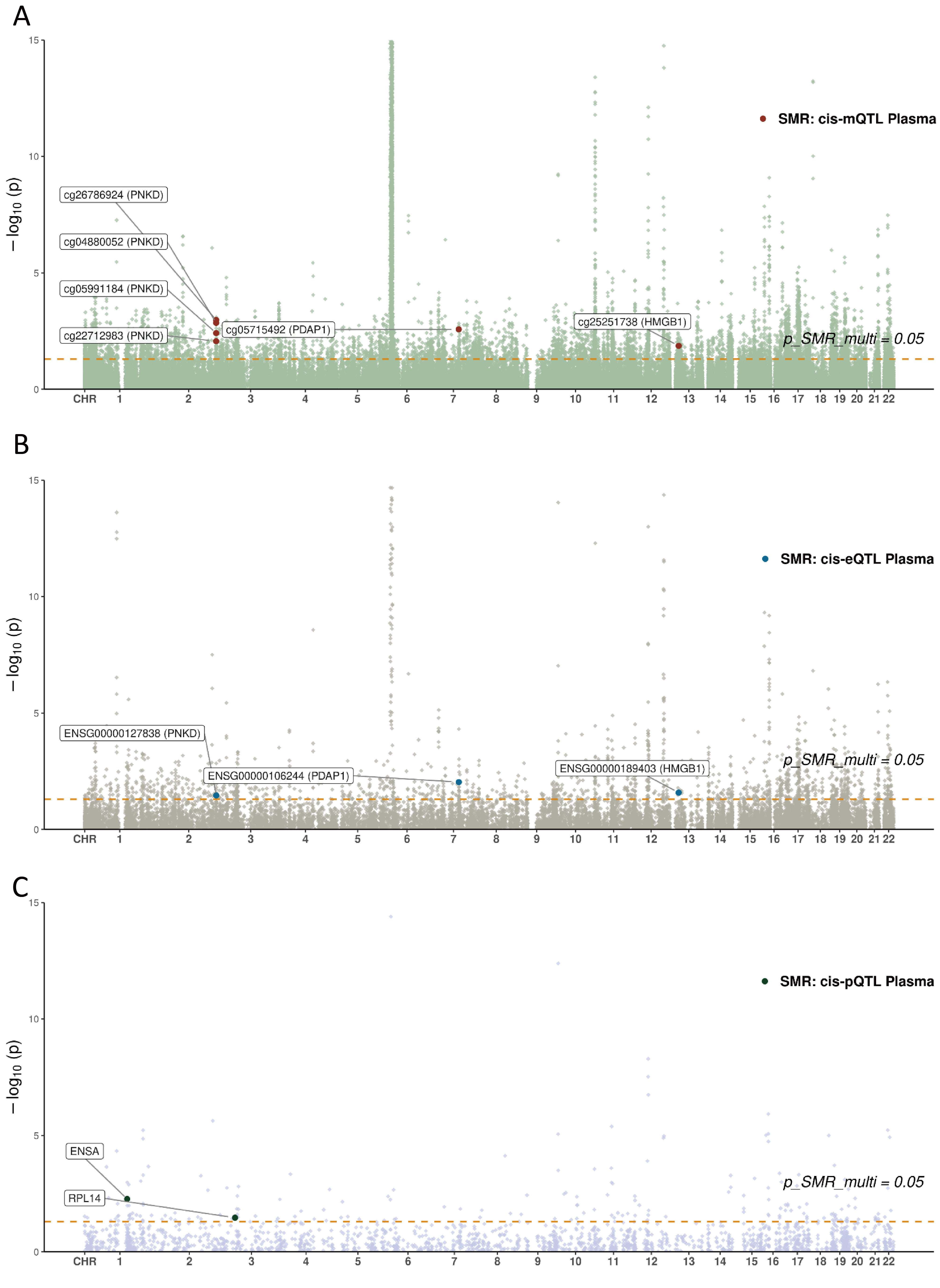

Supplement: Supplementary file 1 — Supporting Information 1 Figure S1: Manhattan plots of SMR associations between lactylation‐related molecular traits and T1D risk in the discovery dataset (GCST90000529). Panels display genome‐wide SMR results based on (A) mQTLs, (B) eQTLs, and (C) pQTLs. Each point represents an SMR association, plotted as −log10(p). The dashed horizontal line indicates the SMR multiple‐testing threshold (p_SMR_multi = 0.05). Selected loci of interest are annotated. [file JDR-2026-9544974-s012.jpg]

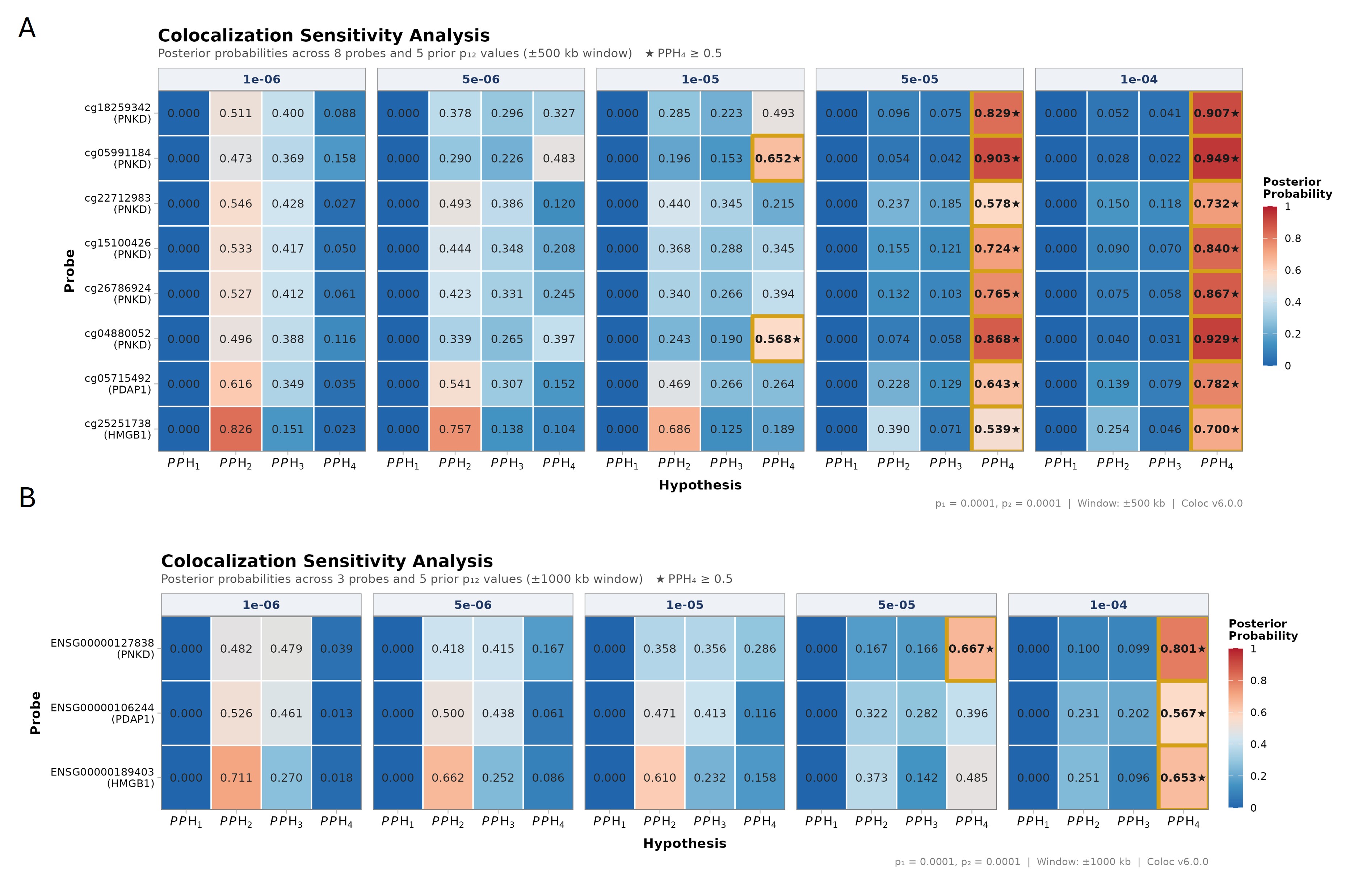

Supplement: Supplementary file 2 — Supporting Information 2 Figure S2: Sensitivity analysis of colocalization results under different p12 priors. (A) Sensitivity analysis of mQTL‐GWAS colocalization results for prioritized CpG sites in PNKD, PDAP1, and HMGB1 using a ±500‐kb genomic window. (B) Sensitivity analysis of eQTL‐GWAS colocalization results for prioritized transcripts in PNKD, PDAP1, and HMGB1 using a ±1000‐kb genomic window. Colocalization analyses were repeated using five p12 priors (1 × 10^−6^, 5 × 10^−6^, 1 × 10^−5^, 5 × 10^−5^, and 1 × 10^−4^). Heatmaps display posterior probabilities for the four coloc hypotheses: PPH1, association with the QTL trait only; PPH2, association with T1D only; PPH3, association with both traits driven by distinct causal variants; and PPH4, association with both traits driven by a shared causal variant. Numerical values represent posterior probabilities. Warmer colors indicate higher posterior probabilities. Cells outlined in gold and marked with an asterisk denote colocalization support (PPH4 > 0.5 and PPH3 < 0.5). Abbreviations: mQTL, methylation quantitative trait locus; eQTL, expression quantitative trait locus; GWAS, genome‐wide association study; T1D, type 1 diabetes; PPH, posterior probability of hypothesis. [file JDR-2026-9544974-s011.jpg]
